# Probiotics in the treatment of acute rotavirus diarrhoea. A randomized, double-blind, controlled trial using two different probiotic preparations in Bolivian children

**DOI:** 10.1186/1471-2334-10-253

**Published:** 2010-08-25

**Authors:** Giuseppe Grandy, Marcos Medina, Richard Soria, Carlos G Terán, Magdalena Araya

**Affiliations:** 1 Paediatric Centre Albina Patiño, Department of Gastroenterology and Nutrition, Department of Infectology. Cochabamba, Bolivia; 2 Albina Patiño Foundation, Cochabamba, Bolivia; 3 Institute of Nutrition and Technology of Foods, University of Chile, Macul 5540, Santiago, Chile

## Abstract

**Background:**

Evidence suggests that probiotics reduce rotavirus diarrhoea duration. Although there are several probiotic strains potentially useful, daily practice is often limited by the type and number of products locally available. In general, information about combined products is scarce. In this study we compare the effect of two probiotic products in the treatment of diarrhoea in children less than 2 years of age.

**Methods:**

A Randomized double-blind controlled clinical trial in children hospitalized for acute rotavirus diarrhoea, in the Paediatric Centre Albina Patino, Cochabamba, Bolivia.

Participants were children aged 1 - 23 months, who were randomly assigned to receive one of three treatments: Oral rehydration therapy plus placebo; Oral rehydration solution plus *Saccharomyces boulardii*; or Oral rehydration solution plus a compound containing *Lactobacillus acidophilus, Lactobacillus rhamnosus, Bifidobacterium longum and Saccharomyces boulardii*. Sample size was 20 per group and the outcomes were duration of diarrhoea, of fever, of vomiting and of hospitalization.

**Results:**

64 cases finished the protocol. On admission, patients' characteristics were similar. Median duration of diarrhoea (p = 0.04) in children who received the single species product (58 hours) was shorter than in controls (84.5 hrs). Comparing children that received the single probiotic product and controls showed shorter duration of fever (18 vs 67 hrs) (p = 0.0042) and the mixed probiotic of vomiting (0 vs 42.5 hrs) (p = 0.041). There was no effect on duration of hospitalization (p = 0.31). When experimental groups were merged, statistical significance of changes increased (total duration of diarrhoea, fever and vomiting P = 0.025, P = 0.025 and P = 0.014, respectively).

**Conclusions:**

Both products decreased the duration of diarrhoea compared to oral rehydration solution alone. This decrease was significant only for the single species product which also decreased the duration of fever. With the multiple species product there was no vomiting subsequent to the initiation of treatment. The quantity of probiotic bacteria needed for optimum treatment of gastroenteritis remains to be determined, particularly when multiple species are included in the product.

Trial registration: ClinicalTrials.gov ID: NCT00981877

Link: https://register.clinicaltrials.gov/prs/app/action/SelectProtocol/sid/S0002653/selectaction/View/ts/2/uid/U0000N04

**Trial Registration:**

Clinical trials NCT ID: NCT00981877

## Background

Acute gastroenteritis is an infectious syndrome that represents the first cause of hospitalization in children. Cohort studies show that nearly all children suffer at least one rotavirus infection before reaching 5 years of age, independent of their socioeconomic status [1]. Below one year of age, rotavirus represents the main etiologic agent, both in developed and developing countries [2-4]. Globally, this agent is responsible for approximately 600.000 deaths per year [2], 82% of which occur in less developed areas. In Latin America, rotavirus gastroenteritis represents 16 to 52% of cases [5] whereas in Bolivia, in 2008 the Health Sentinels System reported that below 5 years of age rotavirus was the main cause of severe gastroenteritis in children, affecting all socioeconomic conditions; thus, rotavirus was responsible for 40% of hospitalizations and 50% of deaths [6]. In 1989, a study by Lopez et al in Cochabamba, Bolivia, described that rotavirus was found in 22.5% of cases admitted for acute diarrhoeal disease [7]. More recently, a study conducted in 2007 also in Cochabamba, confirmed the high frequency (19%) of rotavirus in children admitted for acute diarrhoea [8].

Treatment of diarrhoea basically consists of replacing lost fluids by means of oral rehydration solutions [9,10]; in order to minimize the nutritional impact, treatment aims at shortening the period of fluid losses (diarrhoea and vomiting) and total time of diarrhoea. Although oral rehydration solutions successfully avoid death associated with dehydration and acidosis, they are not effective in shortening the duration of rotavirus-induced diarrhoea and of high fluid losses [11-14]. Testing different strategies to help in this direction, probiotics appear as one of the alternatives currently under discussion [15]. That probiotics may shorten the time of diarrhoea and therefore the time of rotavirus excretion [11,15] is of epidemiological relevance and deserves study.

Although there are several probiotic strains that could be used for treatment, in daily practice we are often limited by the type and number of products locally available. In general, information about combined products is scarce. With this in mind, in this study we compared the efficacy of two commercially available products, one containing *S boulardii *(single species product) and the other combining *L. acidophilus, L. rhamnosus, B. longum *(multiple species product), in children with rotavirus associated diarrhoea.

## Methods

### Design

This was a prospective, double blind, randomized protocol conducted in children 1 to 23 months of age, hospitalized for acute diarrhoea at the Paediatric Centre Albina Patiño (CPAP) between July 2007 and February 2008 in Cochabamba - Bolivia. These children were evaluated for rotavirus, as well as for bacterial pathogens and parasites. Parents received detailed information about the study and those who agreed to participate signed an informed consent. The protocol was reviewed and approved by the Ethics Committee of CPAP; also, we explained the study in detail to the professionals in charge of the patients care such that they would be motivated and willing to follow the protocol strictly.

Operational definition of acute diarrhoea was defined as the presence of at least three bowel movements more than the normal number for the child and/or presence of watery stools per day, plus a latex test positive for rotavirus within 24 hour prior to hospitalization or within 6 hours after hospitalization. Exclusion criteria were Weight/Height (WHO standards, 2006,) [16] at or below -3SD, dehydration >10% (because patients received iv fluids), severe electrolytic imbalance (hypokalemia <3.5 mEq/L, hypernatremia < 145 mEq/L), detection of bacterial and/or parasitic agents of diarrhoea in the stools, detection of other infections (sepsis, pneumonia, urinary infection), diagnosis of immune deficiency, administration of antibiotics, anti diarrheal drugs or probiotics during the 7 days prior to admission to the protocol.

**Figure 1:**
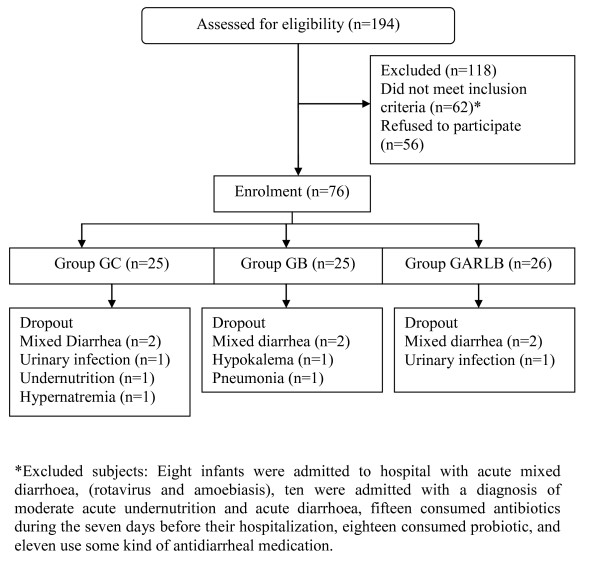
**Flow Diagram**. Flow of participants through trial.

On admission to the study data about full clinical history, physical examination, nutritional status, dehydration, fever, oral tolerance, and stools characteristics, were recorded. After the patient clinically stabilized and maintained hydration for at least 3 hours (within approximately 24 hours), cases were randomized to one of three groups (figure 1): Group GC received oral rehydration solution (ORS) and a placebo, Group GB received ORS plus *S boulardii *and group GARLB received ORS plus the combined probiotic product *L. acidophilus, L. rhamnosus, B. longum *and *S. boulardii *(table 1). Placebo and probiotic products had similar colour and taste. They were administered for 5 days, twice daily, dissolved in 20 ml of water, as indicated by the manufacturer. During the first 48-72 hours the patients remained in hospital; then the attending physician decided when the child was discharged, on the basis of the his/her clinical condition and absence of diarrhoea, vomiting or fever. After discharge the infants were monitored once a day until 5 days of treatment were completed. Controls was performed at the hospital, by the attending physician, recording probiotic intake, frequency and appearance of stools, presence of fever and other relevant clinical features.

**Table 1:** Micro organisms load according to the manufacturer, administration, and main characteristic of probiotic products analyzed.

Group	Microorganisms	Dose (twice daily)	Price ($)*
GARLB	*L. acidophilus*,	6.625 × 10^7 ^lyophilized cells/dose	8.71
	*L. rhamnosus*,	3.625 × 10^7 ^lyophilized cells/dose	
	*B. longum*,	8.75 × 10^6 ^lyophilized cells/dose	
	*S. boulardii*	1.375 × 10^7 ^lyophilized cells/dose	

GB	*S. Boulardii *	4 × 10^10 ^lyophilized cells/dose	11.43

*Price corresponds to complete treatment course at the time of the study.

#### Diet

All children received the same diet; those below 6 months of age maintained breast feeding, adding infant formula (NAN 1^®^, Nestle, Vevey, Switzerland) only when the mother could not be present. After 6 months of age and in addition to breast feeding and formula, infants were offered porridge prepared with chicken, rice, and vegetables (carrots and potatoes).

#### Variables

The study variable was defined as length of diarrhoea (in hours), from admission to the first formed stool; presence of stools and their consistency was checked every four hours, classifying them in liquid, semi-liquid, soft and formed. Secondary variables were vomiting (measured in hours from admission to the last recorded emesis), duration of fever (> 38°C rectal temperature, measured every six hours from admission to discharge). Height was measured in centimetres on admission and discharge; weight was measured every eight hours using a scale with 0.5 g increments and up to 15 kg capacity.

#### Procedures

Blood count included haemoglobin and peripheral white blood count and differential formula; determination of semi quantitative CRP was performed using the commercial kit Humatex^® ^CRP(Human Gessellshaff, Wiesbaden, Germany); sodium, potassium and calcium in plasma using Photometry using Sherwood^® ^Model 410 Classic Flame Photometer Range (Sherwood Scientific Limited, Cambridge, UK); latex test for rotavirus using Rida^® ^Quick Rotavirus/Adenovirus Combi (R-Biopharm AG, Darmstadt, Germany) and parasitological assessment by PAF technique by Lugol method [17]. Stool culture was performed for enteropathogenic, toxigenic, enteroadherent, enteroaggregative, invasive and enterohemorrhagic *E coli*, *Salmonella typhi *and *Shigella sp*, applying routine procedures (Agar base, MacConkey Agar, SS agar and Tetrahionate Broth Base. Bacto Difco^®^, Kansas, USA).

#### Sample size

This was calculated using the data published by Guarino [18], who described the effects of a product that combined three probiotics in children with rotavirus diarrhoea and found that diarrhoea decreased from 120 ± 30 to 96 ± 30 (24 hours decrease); using 0.8 power, 0.05 (one-sided) significance and assuming 24 hours difference between the experimental and control groups the sample size was calculated as 20 cases per group.

### Statistical analysis

This included the comparison between the control and each of the intervention groups and between the two intervention groups, using non parametric Kruskall-Wallis testing for comparison of medians in continuous variables and Mann-Whitney U test. Chi square was applied to categorical variables. An additional analysis compared the control group with the merged intervention groups, using Mann - Whitney U test. Data were processed using Microsoft excel and STATA 1.0

## Results

As shown in the algorithm (figure 1), a total of 194 children below 2 years of age were admitted during the study period; 76 fulfilled the protocol inclusion and exclusion criteria; 12 patients were excluded from analysis: in six, other etiologic agents were found together with rotavirus (amoebas = 4, *Shigella sp*= 2); 2 patients developed E coli associated urinary infection; in one pneumonia was diagnosed; one developed bilateral oedema and kwashiorkor; 2 patients developed severe vomiting for several days and maintained electrolytic imbalance; thus, the final number of patients analyzed was 64. General characteristics of patients excluded were not different from those that completed the study period (data not shown).

Sex, age and characteristics of diarrhoea prior to admission appear in table 2.

**Table 2:** Basal characteristics of groups and secondary outcomes

	GC Group n= 20	GARLB Group n = 23	GB Group n = 21	P value
Boys (%)	9 (45)	15 (65)	12 (57)	0.40*

Age, months (IQR)	11 (8.5)	6 (5)	8 (7)	0.08^†^

Weight, gr. (IQR)	7775 (1769.5)	7652.5 (2765)	7800 (2115)	0.92^†^

Duration(IQR) of diarrhoea before treatment (hours)	48 (24)	72 (36)	48 (48)	0.17^†^

Median hours of hospitalization (IQR)	89.5 (117)	72 (36)	60 (41)	0.31^₤^

N° (%) of children with fever	19 (95)	21 (91)	18 (86)	0.59*

Median (IQR) duration of fever (hours)	67 (60)	48 (36)	18 (53)	0.0042^†^

N° (%) of children vomiting	13 (65)	10 (43.5)	11 (52.4)	0.37*

Median (IQR) duration of vomiting (hours)	42.5 (69.5)	0 (25)	4 (44)	0.041^‡^

Patients' characteristics on admission. Duration of hospitalization, of fever and of vomiting after treatment.

IQR= Intercuartile Range

* Chi square test

† Kruskall - Wallis test

₤ Kruskall - Wallis test, adjusted p value, median comparison between GC and GB groups.

‡ Kruskall - Wallis test, adjusted p value, median comparison between GC and GARLB groups.

Total duration of diarrhoea was significantly shorter in children receiving *S boulardii *(P = 0.04) and the decrease observed in those receiving GARLB was non significant (P = 0.06) (table 3); differences were not significant either between the two intervention groups (table 3). Although the number of children with fever was similar in the three groups, duration of fever was significantly shorter in the group receiving *S boulardii *(as compared to controls) (P = 0.0042), whereas no changes were observed in group GARLB (also compared to controls). In the same way, the number of children with vomiting was not different between the three groups, but group GARLB showed vomiting for a significant shorter time than controls (P = 0.041) (table 2). Diarrhoea, vomiting or other complications were detected after hospital discharge (data not shown). When the merged intervention groups were compared with controls (Table 4), total duration of diarrhoea, fever and vomiting were significantly shorter, (P = 0.025, P = 0.025 and P = 0.014, respectively).

**Table 3:** Principal Outcome

Group	Treatment	Median (IQR) duration (hrs.)	*p value
GARLB	*L. acidophilus, L. rhamnosus, B. longum, S. boulardii *	60 (40)	0.06^†^

GB	*S. boulardii *	58 (41)	0.04^†^

GC	Control group	84.5 (94)	------

Duration of diarrhoea after treatment.

IQR= Intercuartile Range

* p value: comparison of median with control group.

† U Mann - Whitney test.

**Table 4:** Comparison of the merged intervention groups with controls

	Control Group n= 21	Intervention Group* n = 43	p value**
Median hours of hospitalization (IQR)	89.5 (117)	76 (48)	0.13

Median hours of diarrhoea (IQR)	84.5(94)	60(40.5)	0.025

Median (IQR) duration of fever (hours)	67 (60)	46.5 (50.5)	0.025

Median (IQR) duration of vomiting (hours)	42.5 (69.5)	0 (33)	0.014

Duration of diarrhoea, hospitalization, fever and vomiting

IQR= Intercuartile Range

* = Intervention group represents the addition of the two groups receiving probiotic products

**Mann - Whitney U Test

## Discussion

Results show that *S boulardii *diminished the time of diarrhoea by 31.4% and shortened time with fever by 73% (table 3). Children receiving the multiple species product tended to have less time with diarrhoea and no patients vomited after the treatment was started. In previous studies that administered multiple species products similar to the one we used, other authors found a rather more pronounced effect, 30 hours [14,19] and 30-36 hours reduction in diarrhoeal duration [20-23], in comparison with the 26 hours reduction we found. Infants hospitalized in our study were admitted with severe diarrhoea and had intense clinical manifestations in comparison to outpatients with rotavirus diarrhoea; this could explain the less intense results obtained. Although not significant, we consider relevant the trend to diminish time of diarrhoea in the group receiving the multiple probiotic products, because decreasing severity of diarrhoea may help reducing the nutritional impact of the diarrhoeal episode.

Other factors such as poor nutritional status, severe diarrhoea, severe dehydration, should not be confounding variables in this protocol because they were all exclusion criteria. Reports in the literature about children with rotavirus diarrhoea refer mainly to cases managed as outpatients. This study provides evidence that probiotics are also helpful in cases with less than 10% dehydration that require hospitalization. It is worth noting that in this study, despite one day less of diarrhoea, the total length of hospital admissions did not decrease. This was mainly due to requests of mothers and fathers to maintain the child one more day under observation in hospital because, living far from hospital; they feared their child would need to be readmitted to hospital.

Effects of probiotics on vomiting are not clear. Some studies have reported no effects [24] whereas other authors report a significant decrease on time of vomiting [25] or a transitory effect, observed only during some days of the episode [26]. Our results support the effect of probiotics on vomiting, showing decreased time of vomiting in the intervention groups as compared with controls (zero versus 40 hours). However only in the multiple species product-treated group the shorter time of vomiting reached significance. Furthermore, children receiving the single species product had almost 50 hours less fever than the control group. This last feature is in contrast to studies by other authors and also the results of a study conducted in our hospital comparing Nitazoxanide to probiotics, none of which detected less time with fever [27].

This study compared two probiotic products, both readily available in Bolivia, one with a single species of probiotic bacteria and the other with multiple species of probiotic bacteria. The former contains higher total concentrations of bacteria despite having only one species and yielded better results. This raises the question as to whether larger doses of one probiotic bacterial strain are more efficient than multiple species in smaller numbers.

The effect of different probiotic species and strains on diarrhoea is currently well accepted [28-32]; however, the dose required to obtain the best results is less clear. In a recent study by Fang et al [33]*L. rhamnosus *reduced faecal excretion of rotavirus in a dose dependent fashion; authors concluded that the minimal dose required to have a positive effect was at least 6 × 10^8 ^CFU, which coincides with other authors [34]. In a recent metanalysis by Guandalini [31] the recommended dose was at least 5 × 10^9 ^CFU. Other authors found no effect in duration of diarrhoea using 1 × 10^7 ^*L. rhamnosus *[28]. When we analyzed the products used in our study, we found that the mixture of probiotics included a total amount of bacteria of 1.25 × 10^9^; estimating the individual dosing of each probiotics present, they were well below the amount described as effective. The fact that we found positive effects on time of diarrhoea, of vomiting and of fever suggests that the total amount of bacteria present in the product indeed influences the results, but it also suggests that adequate numbers, as it was the case of the single probiotic, yields better results than a mixture in lesser numbers. Discussing the dose provided by each commercial product is relevant because there is evidence suggesting that the effect obtained is dose dependent [32,35], the higher the dose the clearer effect. However, studies of adverse reactions also seem related to probiotic dosing, therefore, the appropriate amounts of bacteria should be established for each probiotic when administered in mixtures, such as that they are best inducing the effect and at the same time are safe for the patient.

## Conclusions

In summary, results of this study support the use of probiotics in treating rotavirus diarrhoea. They would be especially relevant in societies where diarrhoea and malnutrition have high prevalence; decreasing time of diarrhoea, of vomiting and of fever will help diminishing (and/or preventing) malnutrition secondary to acute diarrhoea.

However, studies of adverse reactions also seem related to probiotic dosing, therefore, the appropriate amounts of bacteria should be established for each probiotic when administered in mixtures, such as that they are best inducing the effect and at the same time are safe for the patient.

## Competing interests

The authors declare that they have no competing interests.

## Authors' contributions

GGA conceived of the study, participated in the design of the study, supervision of the research group, participated in the collection data, performed the statistical analysis and drafted the manuscript. MMB participated in the design of the study and drafted the manuscript. RSM participated in the design of the study and performed the statistical analysis. CTM participated in the supervision of the research group, and participated in the collection data. MAQ participated in the coordination and helped to draft the manuscript. All authors read and approved the final manuscript.

## Pre-publication history

The pre-publication history for this paper can be accessed here:

http://www.biomedcentral.com/1471-2334/10/253/prepub
